# Pattern of the Heart Rate Performance Curve in Subjects with Beta-Blocker Treatment and Healthy Controls

**DOI:** 10.3390/jfmk6030061

**Published:** 2021-07-13

**Authors:** Philipp Birnbaumer, Heimo Traninger, Matteo C. Sattler, Andrea Borenich, Peter Hofmann

**Affiliations:** 1 Institute of Human Movement Science, Sport & Health, University of Graz, 8010 Graz, Austria; philipp.birnbaumer@uni-graz.at (P.B.); matteo.sattler@uni-graz.at (M.C.S.); 2 ZARG Centre for Outpatient Rehabilitation, 8021 Graz, Austria; heimo.traninger@pro-heart.at; 3 Department of Production and Operations Management, University of Graz, 8010 Graz, Austria; andrea.borenich@uni-graz.at

**Keywords:** cardiac rehabilitation, exercise prescription, exercise intensity, heart rate deflection point

## Abstract

(1): Heart rate performance curve (HRPC) in incremental exercise was shown to be not uniform, causing false intensity estimation applying percentages of maximal heart rate (HR_max_). HRPC variations are mediated by β-adrenergic receptor sensitivity. The aim was to study age and sex dependent differences in HRPC patterns in adults with β-blocker treatment (BB) and healthy controls (C). (2): A total of 535 (102 female) BB individuals were matched 1:1 for age and sex (male 59 ± 11 yrs, female 61 ± 11 yrs) in C. From the maximum incremental cycle ergometer exercise a first and second heart rate (HR) threshold (Th1 and Th2) was determined. Based on the degree of the deflection (kHR), HRPCs were categorized as regular (downward deflection (kHR > 0.1)) and non-regular (upward deflection (kHR < 0.1), linear time course). (3): Logistic regression analysis revealed a higher odds ratio to present a non-regular curve in BB compared to C (females showed three times higher odds). The odds for non-regular HRPC in BB versus C decreased with older age (OR interaction = 0.97, CI = 0.94–0.99). Maximal and submaximal performance and HR variables were significantly lower in BB (*p* < 0.05). %HR_max_ was significantly lower in BB versus C at Th2 (male: 77.2 ± 7.3% vs. 80.8 ± 5.0%; female: 79.2 ± 5.1% vs. 84.0 ± 4.3%). %P_max_ at Th2 was similar in BB and C. (4): The HRPC pattern in incremental cycle ergometer exercise is different in individuals receiving β-blocker treatment compared to healthy individuals. The effects were also dependent on age and sex. Relative HR values at Th2 varied substantially depending on treatment. Thus, the percentage of Pmax seems to be a stable and independent indicator for exercise intensity prescription.

## 1. Introduction

To induce desired training effects and to apply safe exercise programs, exercise prescription is commonly based on fixed percentages of maximal heart rate (HR_max_) or maximal oxygen consumption (VO_2max_). Usually, intensity ranges, are prescribed between 65% to 85% of HR_max_ or 50% to 75% of VO_2max_ [1]. However, fixed-percentage approaches were shown not to guarantee a uniform load amongst individuals [2,3]. Different metabolic responses, which may vary from over- to under-loading have been prescribed [4]. The inconsistency between intensity domains claims the need for adjustment and new indicators in intensity prescription, proposed by the 2020 position paper from the European Association of Preventive Cardiology (EPAC) [5]. Moreover, individualized prescriptions based on cardiopulmonary exercise tests and individual thresholds such as the first and second ventilatory threshold are recommended [5,6,7].

**Figure 1:**
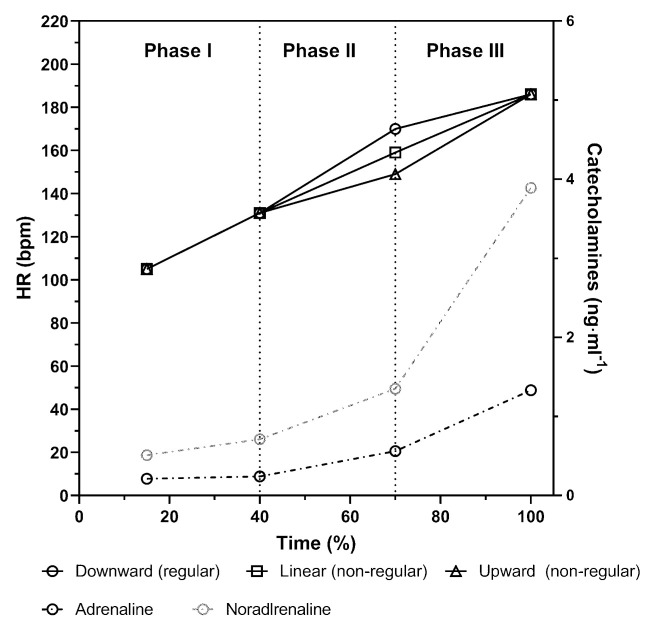
Schematic 3—phase model of the heart rate performance curve (HRPC) during incremental cycle ergometer exercise with downward deflection (regular), linear course or upward deflection (non-regular) (solid black lines) as well as plasma adrenaline and noradrenaline concentrations (dashed and dash-dotted black and grey lines) (modified from Hofmann et al. and Pokan et al. [9,17]).

In practice, simple approaches are usually favored and most exercise tests measure solely electrocardiogram (ECG) based heart rate (HR). Some problems with the use of %HR_max_ for exercise prescription have already been addressed to different HR curve patterns [8] (see Figure 1 for different patterns). These authors showed that the increase of HR during incremental cycle ergometer exercise presented neither a uniform nor a linear pattern which clearly impacts exercise prescription [9]. Most of the young and trained male subjects presented an S-shaped pattern of the heart rate performance curve (HRPC) which was characterized by a distinct flattening of the HR curve at higher intensities (a so-called downward deflection). This course of the HRPC is considered regular [10]. However, in the same study, a significant number of participants showed a linear or even upward deflection [9], which is considered non-regular and was shown to be related to left ventricular function [11]. Recently, we could support these early results by showing this diversity of HRPC patterns in a large cohort of healthy trained and untrained male and female subjects across a wide range of age [12]. Interestingly, age significantly altered the HRPC from regular to non-regular, modulated by maximum exercise performance and sex. These results imply consequences for exercise prescription (e.g., risk of overloading in subjects with non-regular HRPCs) dependent on the above-mentioned variables which has also been critically addressed already by others [2]. Both the downward and upward deflection of the HRPC during incremental exercise is used to determine the so-called HR deflection point (HRDP) [9,13] which is a well-accepted and frequently applied method for threshold determination [14]. Applying the actual standard three Phase-Two threshold model of energy supply, the HRDP corresponds to the second ventilatory or lactate threshold [8,15].

The increase in HR during incremental exercise is strongly related to the increase of plasma catecholamine concentration which drives rate and force of contraction [16]. Pokan et al. [17] showed that the catecholamine response to incremental exercise presented the same pattern than the lactate performance curve in young and trained healthy male subjects, however, the pattern of the HRPC was not related to the catecholamine response in this study (Figure 1). Despite such a similar catecholamine response we could show that the patterns of the HRPC were significantly influenced by a β1-receptor antagonist application [18]. This led to the conclusion that the β1-receptor sensitivity or density are key regulators of the pattern of the HRPC. Reduced β1-receptor sensitivity, either induced by chronic (over) stimulation due to increased sympathetic activity [19] or antagonist treatment, blunts HR increase at rest and submaximal exercise. The reduced sensitivity typically causes inverted HRPCs with upward deflection due to the higher catecholamine effects reaching maximal exercise [18,20] (Figure 1). In a recently published study including 2980 men and 1944 women we could show that the prevalence of HRPC with upward deflection increased from 20 to 70 years (respectively 80 yrs in women) both in men (10–43%) and women (9–30%). This trend might be caused by a decrease in β1-receptor sensitivity with increasing age [21,22,23]. Therefore, the decline in β1-receptor sensitivity is suggested to be the main factor influencing the HRPC deflection pattern. 

Selective or non-selective β-receptor antagonist medication is a standard prescription in cardiology significantly increasing survival when applied long-term [24]. This medication reduces HR and blood pressure at rest and during exertion by a competitive inhibition of the β-receptors counteracting the driving force of stress induced catecholamines [25]. In incremental exercise, β-blocker administration was shown to significantly reduce HR at all workload levels but most at submaximal exercise such as the first and second thresholds in healthy individuals [18,26] as well as patients with cardiovascular disease [27]. 

As most patients with cardiovascular diseases are on β-adrenoceptor antagonist treatment [28] the above prescribed effects regarding exercise prescription need to be concerned. Individuals receiving a β-blocker are supposed to show a higher number of non-regular HRPCs compared to individuals without such a treatment. Studying the expected differences in the HRPC (and the prevalence of non-regular patterns) due to β-blocker intake is highly relevant for the accuracy of exercise prescription [29]. Therefore, the aim of this study was to investigate age and sex related differences in the distribution of HRPC patterns in adults receiving a β-adrenoceptor antagonist treatment and healthy controls. A secondary aim was to explore age and sex related differences in performance variables (i.e., HR_max_, P_max_) between adults receiving beta blocker treatment and healthy controls.

## 2. Materials and Methods

### 2.1. Study Population

In this retrospective, observational study, HR and performance data from maximal cycle ergometer tests from 1070 individuals were analyzed. The groups were composed of 433 male (59 ± 11 yrs) and 102 female (61 ± 11 yrs) individuals treated with β-adrenoceptor antagonists (BB) who were exactly age- and sex-matched 1:1 with 433 male (59 ± 11 yrs) and 102 female (61 ± 11 yrs) healthy individuals in the control group (C). Exercise tests were carried out for performance diagnostics, health preventive or medical reasons between 2004 and 2017 in a cardiology center. The study was approved by the Ethics committee of the local university (GZ. 39/70/63 ex 2016/17). Individuals gave their written consent that their data may be used anonymously for scientific purposes. Only tests with no outliers (non-physiological HR deviation) or missing of HR recordings were included in the analyses. The test protocol was uniform such as to obtain maximal workload within 12–15 min. The protocols for BB and C were independent from age, sex and performance. All ergometer tests in C started at 20 W and workload was increased by 20 W increments every minute up to voluntary exhaustion. In BB, all ergometer tests started at 10 W and workload was increased by 10 W increments every minute up to voluntary exhaustion. In BB, no detailed information about the individual dosing were available for our analysis. 

### 2.2. Assessment of Heart Rate and Performance Data

HR variables were provided as mean values for each single load step (e.g., HR for 20 W, 30 W). Data were analyzed via Vienna CPX-Tool (University of Vienna, Austria) to determine maximal and submaximal HR and performance markers as well as the degree and the direction of the HRPC. To detect a first (Th1) and second threshold (Th2) (equivalent to HRDP) from HR, two regions of interest were defined and consistently applied. Multiple linear regressions were performed for the detection of Th1 between the start of exercise and 66% of P_max_ and for the detection of Th2 between 40% P_max_ and P_max_. The degree and direction of the deflection of the HRPC (k_HR_ = (k1 − k2)/(1 + k1 × k2)) was calculated from the slopes of two tangents of a second-degree polynomial function fitted between 40% P_max_ and P_max_. Because Vienna CPX-Tool subtracts the slopes of the tangents (k1 and k2) in a different order compared to earlier analyses [9,11,12] we changed the algebraic sign from negative to positive and vice versa to be consistent with previous studies. Based on the k_HR_ values HRPC´s were categorized as regular HRPC (downward deflection) for k_HR_ > 0.1 and as nonregular HRPC (linear course and upward deflection) for k_HR_ < 0.1 according to Pokan et al. [11] (Figure 1).

### 2.3. Statistical Analysis

Descriptive statistics are shown according to their distribution as mean ± standard deviation (SD) or median (interquartile range (IQR)). Data were tested for normal distribution by means of Shapiro–Wilk test. To compare BB and C within male and female individuals, a student *t* test or Mann–Whitney-U test was used. A multivariable logistic regression model was calculated to evaluate the relationship between β-blocker treatment and the presence of non-regular HRPCs (coded as 1 = non-regular, 0 = regular). In this model, we included treatment (0 = C, 1 = BB), sex (0 = male, 1 = female), age (continuous), age^2^ and the following interactions of interest as explanatory variables: sex × treatment, age × treatment, age^2^ × treatment. We added a quadratic term of age because it better reflected the shape of the relationship. Moreover, a non-linear relationship was also indicated by a significant interaction between age and log(age) (*p* = 0.001). Additionally, two multivariable linear regression models were calculated to evaluate the relationship between β-blocker treatment and HR_max_ and P_max_, respectively. Here, we included age, sex and the following interactions of interest as explanatory variables: sex × treatment, age × treatment. Age was mean cantered before running the analyses. The assumptions of linear and logistic regression analyses were checked and considered to be met (i.e., after the transformation of age). Please note the main effects of age and sex on HRPC were not addressed in this study. Results are expressed using odds ratios (OR), unstandardized regression coefficients (B) and 95% confidence intervals (CI) for explanatory variables as well as R^2^ for the overall model. Finally, to examine age-related associations between β-blocker treatment and submaximal HR and performance variables, data were first categorized into four age groups starting at ≤50 yrs up to >70 yrs. Then, separate 4 × 2 ANOVAs with post hoc Tukey´s multiple comparison tests were applied for males and females. All statistical analyses were performed in SPSS 26 (IBM Corporation, Armonk, NY, USA). Graphical representations were created with Prism 8.0 (GraphPad, San Diego, CA, USA). Statistical significance was considered as *p* < 0.05.

## 3. Results

The mean age of the study population was 59 ± 11 yrs in male (m) and 61 ± 11 yrs in female (f). Body mass index (BMI) ranged from 18.21 to 44.68 kg/m^2^ in males and from 16.36 to 43.25 kg/m^2^ in female individuals. In BB, 82% male and 84% female individuals had a selective β1-blocker and 18% and 16%, respectively—a nonselective blocker. Determination of Th1 and Th2 was successful in all cases except for two. P_max_ and performance at Th1 and Th2 as well as HR_max_ and HR at Th1 and Th2 were significantly lower in BB compared to C for both male and female individuals. In addition, relative performance values were significantly different between BB and C in male and female and ranged overall between 37.5–42.1% at Th1 and 69.8–70.8% at Th2. Absolute HR difference between C and BB at Th1 were 6 ± 16 bpm (m) and 8 ± 15 bpm (f) and at Th2 10 ± 16 bpm (m) and 13 ± 15 bpm (f), i.e., HR values at thresholds were lower in BB compared to C. Mean %HR_max_ at Th1 and Th2 was significantly lower in BB compared to C in male and female. However, the difference between BB and C was smaller at Th1 (m: 1.7 ± 9.6%, f: 2.1 ± 8.8%) compared to Th2 (m: 3.6 ± 8.5%, f: 4.7 ± 7.5%) (Table 1). 

**Figure 2:**
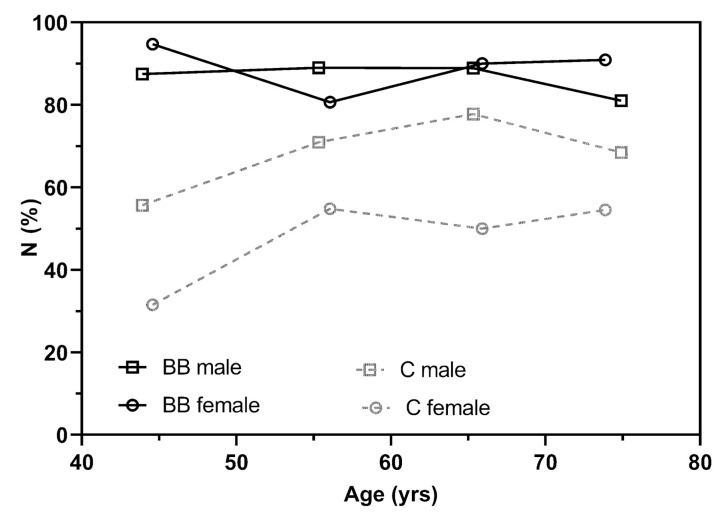
Age related percentage of non-regular HRPCs in male and female individuals with (BB) and without (C) β-blocker treatment.

The number of non-regular HRPCs was higher in BB compared to C (m: 378 vs. 299, *p* < 0.001; f: 90 vs. 50, *p* < 0.001). Whereas the number of regular HRPC was higher in C compared to BB (m: 134 vs. 55, *p* < 0.001; f: 52 vs. 12, *p* < 0.001). The number of non-regular curves for BB and C, dependent on sex and age, are shown in Figure 2.

The logistic regression model (Table A1 in Appendix A), χ^2^ (7, N = 1070) =111.07, *p* < 0.001, Nagelkerke´s R^2^ = 14.8 revealed that the odds of having a non-regular HRPC in an incremental cycle ergometer test were higher among BB than C but the effect was dependent on sex (OR _interaction_ = 2.77, 95% CI: 1.24–6.19, *p* = 0.013) and age (OR _interaction_ = 0.97, 95% CI = 0.94–0.99, *p* = 0.015). The odds of having a non-regular HRPC due to β-blocker treatment were 2.8 times higher among average-aged females than males (females: OR = 7.65, 95% CI: 3.57–16.39, *p* < 0.001; males: OR = 2.76, 95% CI: 1.79–4.26, *p* < 0.001). 

Moreover, the effect of β-blocker treatment on having a non-regular HRPC decreased with older age. For example, the odds of having a non-regular HRPC in BB versus C were OR = 4.04 (95% CI: 2.57–6.34, *p* < 0.001), OR = 2.76 (as above), and OR = 2.12 (95% CI: 1.34–3.34, *p* = 0.001) for 50-years-, 59-years- (mean of the sample) and 70-years-old male individual, respectively (females: OR_50-years_ = 11.19, 95% CI: 5.07–24.72, *p* < 0.001; OR_59-years_ = 7.65, as above, OR_70-years_ = 5.86, 95% CI: 2.76–12.52, *p* < 0.001).

**Figure 3:**
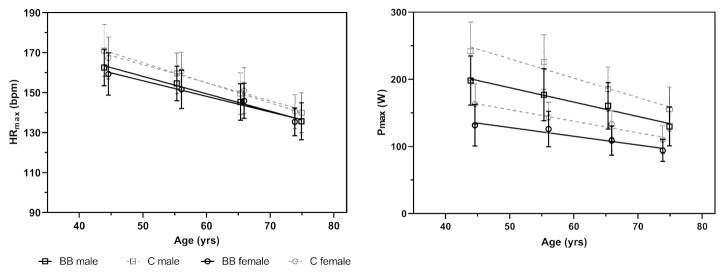
Age related decline of mean maximum heart rate (HR_max_) and power output (P_max_) in male and female individuals with (BB) and without (C) β-blocker treatment.

Figure 3 shows the mean values for HR_max_ and P_max_ categorized in four age-groups for male and female individuals.

The linear regression model for HR_max_ (Table A2), F(5, 1064) = 286.01, *p* < 0.001, R^2^ = 0.57, revealed that, HR_max_ of the incremental exercise test, of an average-aged, male BB was 5.46 (95% CI: 4.24–6.68, *p* < 0.001) bpm less compared to C. This effect was not dependent on sex but age, indicating a decrease in the difference between BB and C among older age (B_interaction_ = 0.17, 95% CI: 0.06–0.27, *p* = 0.001). For example, HR_max_ was, on average, 6.92 (95% 5.40–8.45, *p* < 0.001), 5.46 (as above) and 3.62 (95%CI: 1.97–5.27, *p* < 0.001) bpm lower for 50-years-, 59-years- and 70-years-old male BB, respectively.

The linear regression model for P_max_ (Table A3), F(5, 1064) = 276.21, *p* < 0.001, R^2^ = 0.57, showed that, on average, BB had lower maximum power than C but the magnitude of the difference was dependent on sex and age. When receiving β-blocker treatment, maximum power was reduced by 37.53 W (95% CI: 32.93–42.13, *p* < 0.001) and 23.34 W (−37.53 + 14.19; 95% CI: 13.84–32.84, *p* < 0.001) for an average-aged male and female individual, respectively. These differences were decreasing with older age (B_interaction_ = 0.83, 95% CI: 0.45–1.21, *p* < 0.001). For example, 50-years-, 59-years- and 70-years-old male BB showed, on average, 44.88 W (95% CI: 39.14–50.63, *p* < 0.001), 37.53 W (as above) and 28.31 W (95% CI: 22.09–34.54, *p* < 0.001) lower maximum power compared to C (females: B_50-years_ = 30.69, 95% CI: 20.38–41.01, *p* < 0.001; B_59-years_ = 23.34 W, as above; B_70-years_ = 14.12 W, 95% CI: 4.00–24.25, *p* = 0.006).

ANOVAs revealed significantly lower absolute power output at Th1 and Th2 in male BB compared to C in all four age groups (Table 2). However, relative performance values varied less between BB and C and were not significantly different for Th1 and in two age groups for Th2. Absolute HR values were significantly lower at Th2 in BB, and difference decreased from about 13 bpm in the age group < 50 yrs to 7 bpm in the age group > 70 yrs. %HR_max_ was not statistically different between male BB and C, but values were consistently 2.5 to 4.4% lower in BB. In female individuals, no significant differences at all were found between BB and C, although absolute submaximal HR and performance values were consistently lower in BB. Comparable to male individuals, the HR difference decreased from the youngest to the oldest age group from about 12 to 4 bpm at Th1 and 18 to 8 bpm at Th2. %P_max_ at Th1 and Th2 were comparable to the male sample and varied less between groups.

## 4. Discussion

Our study showed that individuals receiving a β-blocker treatment had usually more than two times higher odds of having a non-regular HRPC (including both linear time course and upward deflection), compared to healthy individuals. However, the odds were dependent on sex and age, indicating higher odds for females and decreasing odds among older individuals. Maximum heart rate as well as maximum power was reduced in BB compared to C, but the effect decreased with higher age. 

Overall, we found 87% compared to 69% non-regular curves in male BB compared to C and 88% compared to 49% in female individuals. The number of non-regular curves in male and female BB was high independent of age, but increased with age in C. The matched healthy group was a sub-sample of a previous investigation, and results are in line with our earlier findings [12] and Hofmann et al. [9], who presented a significantly lower number of only 14% of non-regular curves in a group of 227 young, healthy and trained male subjects. Regarding the number of non-regular curves in BB, no comparative studies were found. However, β-blocker administration was already shown to change the direction of the HRPC from a regular downward deflection to non-regular curve patterns in healthy individuals [18,26]. In particular, Hofmann et al. [18] even showed, that these changes were significantly related to the degree of the deflection in participants randomly receiving placebo or selective β1-adrenoreceptor antagonist. The ”more regular” the curves were in placebo conditions, the greater was the change in β-blocker treatment, whereas non-regular patterns were not affected. Therefore, the higher number of non-regular curves in the present study is consistent with previous literature.

Differences in receptor sensitivity were thought to be a cause for varying responses of the HRPC. In subjects with a regular HRPC, β1-adrenergic receptors were suggested to be sensitive to catecholamines at low intensity levels (Phase 2), leading to a proportional HR increase. At high intensity above Th2 (Phase 3), receptors saturate, and HR increase is damped. This is suggested to cause the flattening of the HRPC, resulting in a regular HRPC [18]. Contrary in subjects with non-regular curves the receptor sensitivity was suggested to be lower, or receptors are blocked due to β-blocker administration. This leads to a blunted HR increase between Th1 and Th2 (Phase 2) (see Figure 1), were catecholamine levels continuously increase but are still moderate. Above Th2 (Phase 3) HR increases disproportionally due to exponentially increasing catecholamine levels [18]. Therefore, the considerably higher number of non-regular HRPCs in the BB group in our study is caused by the β-blocker treatment, especially in the younger subjects. As ageing is associated with a decreased receptor sensitivity [21] and women were shown to have a higher sensitivity compared to men [30], changes in the odds among older individuals and sex can be addressed to changes in the receptor sensitivity. In our previous study we already showed, that women show less non-regular curves compared to men and that the number of non-regular HRPC´s increase with increasing age in healthy individuals [12]. Therefore, the HRPC pattern from BB and C are thought to become more similar with increasing age due to receptor sensitivity decreases with age.

These alterations in the HRPC also influenced the submaximal HR markers, where absolute and %HR_max_ at Th1 and Th2 were found significantly lower in BB compared to C. This was already shown for patients, with and without β-blocker treatment [27], as well as for healthy individuals who randomly received β-blocker or placebo [18,26,31]. The reduced HR at maximal and submaximal intensities in individuals receiving a β-blocker can be addressed to the negative chronotropic effect as desired [25,32]. In our study, the HR differences between BB and C were found between 6 to 8 bpm at Th1 and 10 to 13 bpm at Th2. These values are markedly smaller compared to values of other studies which showed larger differences (Th1: 19 to 26 bpm, Th2: 22 to 37 bpm) [18,26,27,31]. The smaller differences between groups in our study could be due to differences in dosing. However, no information about the dosing was available in the current study. However, a minimum dosage to avoid side-effects during long-term treatment might be explanatory. A further reason might be the higher mean age of our study population. Decreasing maximum HR with increasing age [33] reduces the amplitude of HR from rest to maximal exercise, theoretically effecting the absolute difference between groups. Furthermore, aging was associated with reduced β-adrenergic receptor sensitivity [21] which possibly leads to smaller differences in the HR response to exercise between individuals with and without β-blocker with increasing age. Both age dependent reasons can be supported by our data, were the difference in HR at maximal and submaximal values is shown to decrease between groups with increasing age (Table 2). In terms of HR_max_, values in BB were approximately 5 bpm lower compared to C. The difference was greater in younger compared to older age. Hence, increasing age reduces the difference between individuals with and without β-blocker treatment. Considering that age reduces the receptor sensitivity, older people seem to more likely respond like individuals on β-blocker treatment. However, comparable studies showed larger differences in HR_max_ of about 19 to 38 bpm between patients [27] and healthy individuals with and without β-blocker treatment [18,26,27,31].

Regarding relative values, we found significantly smaller %HR_max_ at Th1 and Th2 in BB. Values at Th2 were below the common upper limit of 85% HR_max_ in BB in 93% and 86%, respectively in C in 79% and 56% of all cases in male and female individuals. Therefore, both groups are overestimated with respect to the upper limit of exercise intensity if based on such a standard prescription. Furthermore, individuals with β-blocker treatment presented a lower %HR_max_ at Th2 (as well as Th1) compared to healthy individuals. Although the differences between BB and C were small, we can show for the first time a different heart rate response during an incremental cycle ergometer exercise in most of the individuals receiving a β-blocker therapy.

Overall, performance in male and female BB was normal with respect to age predicted P_max_ and C were slightly better trained. Interestingly, %P_max_ at Th1 and Th2 was found very similar in BB and C, both in females and males. The percentage ranged between 37.5 and 42.1% at Th1 and between 69.8 and 70.8% at Th2. These values are highly comparable to results from young healthy subjects were a percentage of 38.8 and 72.0% was shown for the first and second lactate threshold [29]. Based on these findings, intensity prescription by means of %P_max_ seems to be more generalizable compared to relative heart rate values [29]. A more individualized prescription and increased consideration of exercise intensity prescription including %P_max_ was recommended by EPAC [5]. Therefore, exercise prescription via fixed percentage of HR_max_ should be avoided and individual thresholds or at least percentages of P_max_ are recommended, especially in individuals on β-blocker treatment in order to avoid overloading [7].

The detection of a first threshold of HR within a fixed region of interest revealed a mean %HR_max_ at Th1 of healthy individuals comparable to HR values of the first ventilatory threshold from the literature [26]. Only a few studies examined the determination of a first heart rate threshold/turn point by mathematical models and showed no significant difference compared to a gold standard detection (e.g., AT-Wassermann). Therefore, the detection of changes in response patterns of HR is suggested to be adequate and promising for the detection of a first threshold in a three-phase model [34,35]. Although this method is yet not very common, this might be of interest for practice.

The present study is not without limitations. One limitation is the lack of any medical diagnosis in C as well as in BB. Due to the advanced age, the likelihood that also C have undiagnosed health conditions is high. Therefore, we cannot exclude that the deflection of the HRPC has been influenced by the consequences of any cardiovascular disease or events [36]. This also refers to BB, where a medical diagnosis is obvious. Furthermore, we do not have any information regarding the β-blocker dosing, which does not allow to discuss any dose-response effects of β-blocker administration on the deflection of the HRPC. However, the presented data reflect the practical situation in rehabilitation and secondary prevention and did not underly any controlled study setting, possibly explaining the smaller differences between groups in this study. Further, also limiting is that performance in BB was lower compared to C, although individuals were matched 1:1 for age and sex. Based on our earlier findings, this might influence the pattern of the HRPC due to the fact that individuals with lower performance were shown to present a higher number of non-regular curves [12]. Regarding exercise prescription, the prescription via %VO_2max_ is an even more common and accepted model beside %HR_max_. Due to the proportional relationship of VO_2_ and performance, exercise prescription based on %VO_2max_ might be more accurate compared to %HR_max_ although not measured in our study. However, Hofmann et al. [18] showed, that compared to %HR_max_, %VO_2max_ was not affected by a selective β-blocker. In the future, investigations regarding the dosing, the influence of selective and non-selective β-blocker application, including spirometric data, are necessary to better understand the implications of β-blocker treatment for exercise prescription. Nevertheless, our study prescribes relevant information regarding age and sex-dependent influences of β-blocker treatment on HR changes during a standardized incremental exercise and its consequences for exercise prescription.

## 5. Conclusions

The HR increase to incremental cycle ergometer exercise is neither uniform nor linear and differs considerably between individuals. BB showed higher odds of having a non-regular HRPC and lower HR_max_ and P_max_ compared to C. However, the effects were modified by age and sex. Relative HR values at the Th2 were not constant but varied substantially with β-blocker treatment, questioning the validity of fixed values for exercise prescription. Therefore, a generalization in terms of exercise prescription via fixed percentage of HR_max_ is not recommended and misclassifications and overestimation of upper limits are very likely in individuals with β-blocker treatment. Interestingly %P_max_ seems to be a good trade off to provide valid estimations about exercise intensity, even in patients with β-blocker treatment and supports the request in the actual guidelines to apply such an easy tool [5].

## Figures and Tables

**Table 1:** Anthropometrics and characteristics of the incremental cycle ergometer tests in male and female individuals with (BB) and without (C) β-blocker treatment.

	MALES				FEMALES			
Variables	C *n* = 433	BB *n* = 433	*p*	d/r	C *n* = 102	BB *n* = 102	*p*	d/r
Age (yrs)	59.0 ±10.9	59.0 ±10.9	1.0 ^a^	0.00	60.7 ±10.7	60.7 ±10.7	1.0 ^a^	0.00
BMI (kg/m^2^)	26.2 (4.0; 20.8)	26.8 (4.8; 25.1)	0.003 ^b^	0.10	24.6 (5.3; 18.8)	25.7 (5.6; 26.9)	0.116 ^b^	0.11
BM (kg)	83 (15; 117)	84 (18; 92)	0.121 ^b^	0.05	65 (12; 54)	69 (17; 83)	0.05 ^b^	0.14
P_max_ (W)	200 (60; 280)	170 (60; 210)	<0.001 ^b^	0.37	140 (40; 160)	110 (40; 130)	<0.001 ^b^	0.37
HR_max_ (bpm)	155 (20; 80)	150 (18; 67)	<0.001 ^b^	0.18	154.7 ±13.8	148 (16; 61)	<0.001 ^b^	0.27
P (W)								
Th1	76.4 (21.8; 105.5)	64.5 (19.1; 79.1)	<0.001 ^b^	0.33	50.9 (12.7; 56.4)	46.4 (12.7; 47.3)	<0.001 ^b^	0.29
Th2	145.5 (41.8; 210.9)	119.1 ±30.0	<0.001 ^b^	0.35	96.9 ±20.3	77.7 (28.4; 95.5)	<0.001 ^b^	0.40
HR (bpm)								
Th1	98.1 (15.2; 61.9)	93.4 (14.7; 67.7)	<0.001 ^b^	0.24	106.4 ±10.9	97.6 (12.6; 55.3)	<0.001 ^b^	0.37
Th2	124.1 (19.3; 78.1)	115.1 (14.6; 77.0)	<0.001 ^b^	0.33	129.9 ±14.1	116.9 ±10.2	<0.001 ^a^	0.93
P as % P_max_								
Th1	37.3 (5.0; 9.9)	39.5 (4.2; 14.2)	<0.001 ^b^	0.30	39.4 (3.9; 8.3)	42.1 ±2.8	<0.001 ^b^	0.47
Th2	69.31 (5.1; 7.6)	71.7 (5.1; 7.1)	0.009 ^b^	0.9	72.7 (4.5; 8.3)	68.2 (5.2; 7.1)	0.006 ^b^	0.19 *
HR as % HR_max_								
Th1	63.4 (8.5; 37.6)	62.1 (9.2; 41.5)	0.001 ^b^	0.11	68.9 ±5.0	66.8 ±7.0	0.016 ^a^	0.34
Th2	80.8 ±5.0	77.2 ±7.3	<0.001 ^b^	0.55	84.2 (6.5; 19.7)	79.2 ±5.1	<0.001 ^b^	0.45
k_HR_	−0.4 (1.3; 10.8)	−1.2 (1.32; 10.3)	<0.001 ^b^	0.36	0.1 ±1.0	−1.0 ±1.0	<0.001 ^a^	−1.00

BMI, body mass index; BM, body mass; P_max_, maximum power output; HR_max_, maximum heart rate; P, power output at Th1 and Th2, first and second threshold; HR, heart rate at Th1 and Th2; k_HR_, degree of the deflection of the heart rate performance curve; d, Cohen’s d prescribing the effect size for normal distribution; r, effect size for not normal distributed data (r = z/√n); a, parametric *t*-test; b, Mann–Whitney-Test. * *p* < 0.05.

**Table 2:** Characteristics of the incremental cycle ergometer test in male and female individuals with (BB) and without (C) β-blocker treatment in four representative age groups shown as mean (±SD).

**MALES** ***n* = 433**		**≤50 yrs**	**51–60 yrs**	**61–70 yrs**	**>70 yrs**
*n*		176	155	117	73
P Th1 (W)	C	87.8 ± 15.6	83.1 ± 15.0	69.9 ± 11.1	60.7 ± 11.9
	BB	75.21 ± 14.0 *	68.5 ± 13.7 *	62.5 ± 11.4 *	52.3 ± 9.3 *
P Th2 (W)	C	169.7 ± 31.6	158.0 ± 30.2	129.2 ± 22.8	109.7 ± 22.5
	BB	139.9 ± 26.9 *	125.0 ±2 7.4 *	113.3 ± 25.1 *	90.7 ± 20.2 *
%P_max_ Th1 (%)	C	36.4 ± 2.5	36.9 ± 2.4	37.9 ± 2.5	39.2 ± 2.3
	BB	38.0 ± 2.4	39.0 ± 2.9	39.3 ± 2.9	40.9 ± 3.2
%P_max_ Th2 (%)	C	70.2 ± 2.6	69.9 ± 2.5	69.7 ± 2.5	70.7 ± 2.5
	BB	70.5 ± 2.5	70.7 ± 2.5 *	70.6 ± 2.6 *	70.0 ± 2.5
HR Th1 (bpm)	C	106.5 ± 11.2	100.2 ± 11.1	96.3 ± 10.6	94.2 ± 11.3
	BB	99.0 ± 11.4	94.8 ± 10.5	90.3 ± 9.3	90.7 ± 10.2
HR Th2 (bpm)	C	139.1 ± 13.8	128.6 ± 11.4	119.4 ± 10.2	114.6 ± 10.0
	BB	126.1 ± 11.2 *	118.9 ± 8.9 *	111.6 ± 8.8 *	107.7 ± 9.6 *
%HR_max_ Th1 (%)	C	62.3 ± 5.4	62.8 ± 6.2	64.5 ± 6.9	67.5 ± 7.8
	BB	60.9 ± 6.2	61.4 ± 6.6	61.2 ± 10.2	67 ± 7.4
%HR_max_ Th2 (%)	C	81.3 ± 4.8	80.5 ± 4.6	79.9 ± 5.3	82.0 ± 5.4
	BB	77.6 ± 5.2	77.0 ± 5.0	75.7 ± 11.1	79.5 ± 7.4
k_HR_	C	−0.1 ± 1.1	−0.3 ± 1.0	−0.5 ± 1.2	−0.3 ± 1.4
	BB	−1.0 ± 1.0	−1.1 ± 1.1	−1.2 ± 1.0	−0.9 ± 1.5
**FEMALES** ***n*** **= 102**		**≤50 yrs**	**51–60 yrs**	**61–70 yrs**	**>70 yrs**
*n*		19	31	30	22
P Th1 (W)	C	62.6 ± 9.6	56.1 ± 9.4	52.7 ± 7.2	44.7 ± 8.7
	BB	53.3 ± 11.4	51.3 ± 8.9	45.7 ± 7.8	41.5 ± 6.2
P Th2 (W)	C	115.5 ± 19.5	101.0 ± 16.2	94.7 ± 14.3	78.1 ± 15.9
	BB	90.8 ± 20.6	89.0 ± 19.4	75.4 ± 15.3	65.7 ± 11.3
%P_max_ Th1 (%)	C	38.6 ± 2.3	39.2 ± 2.1	39.7 ± 2.2	40.6 ± 1.2
	BB	40.9 ± 2.8	41.1 ± 2.6	42.3 ± 2.3	44.4 ± 2.0
%P_max_ Th2 (%)	C	71.0 ± 2.5	70.5 ± 2.4	71.0 ± 2.3	70.8 ± 2.3
	BB	69.1 ± 2.3	70.7 ± 2.6	69.4 ± 2.4	69.9 ± 2.4
HR Th1 (bpm)	C	112.8 ± 11.2	109.6 ± 10.4	103.5 ± 9.9	100.1 ± 7.4
	BB	101.2 ± 10.2	101.5 ± 13.5	94.7 ± 6.9	96.7 ± 8.2
HR Th2 (bpm)	C	142.0 ± 13.5	134.1 ± 11.9	126.4 ± 11.1	118.1 ± 9.2
	BB	123.7 ±8.1	121.3 ± 10.6	113.1 ± 7.2	110.2 ± 8.3
%HR_max_ Th1 (%)	C	67.5 ± 5.2	68.2 ± 4.7	68.6 ± 5.1	71.3 ± 4.5
	BB	67.5 ± 5.2	68.2 ± 4.7	68.6 ± 5.1	71.3 ± 4.5
%HR_max_ Th2 (%)	C	84.8 ± 4.4	83.4 ± 4.1	83.8 ± 4.4	84.0 ± 4.4
	BB	77.9 ± 6.5	80.0 ± 4.5	77.6 ± 4.3	81.4 ± 4.5
k_HR_	C	0.41 ± 1.12	−0.07 ± 0.86	0.12 ± 1.06	0.05 ± 1.07
	BB	−0.98 ± 0.90	−1.06 ± 0.98	−1.05 ± 0.99	−1.02 ± 0.97

*n*, Number of tests; P, power output at Th1 and Th2, first and second threshold; %P_max_ Th1 and Th2, P at Th1 as a percentage of maximum power output; HR Th1 and Th2, heart rate at Th1 and Th2; %HR_max_ Th1 and Th2, HR at Th1 and Th2 as a percentage of maximum heart rate; k_HR_, degree of the deflection of the heart rate performance curve; * *p* < 0.05.

## Data Availability

The data presented in this study are available on request from the corresponding author. The data are not publicly available due to hospital confidentiality.

## Appendix A

**Table A1:** Results of the main logistic regression model to evaluate sex and age-related differences in heart rate performance curves (HRPC) dependent on medication.

	B (SE)	OR (95% CI)	*p*
β-blocker	1.015 (0.221)	2.761 (1.788, 4.261)	<0.001
Age	0.022 (0.009)	1.022 (1.005, 1.040)	0.01
Age^2^	−0.002 (0.001)	0.998 (0.997, 0.999)	0.001
Female	−0.914 (0.228)	0.401 (0.256, 0.628)	<0.001
Female × β-blocker	1.019 (0.410)	2.771 (1.239, 6.194)	0.013
Age × β-blocker	−0.34 (0.014)	0.966 (0.940, 0.993)	0.015
Age^2^ × β-blocker	0.001 (0.001)	1.001 (0.999, 1.003)	0.331

B (SE), unstandardized regression coefficients (standard error); OR, odds ration; CI, 95% confidence interval; logistic regression model: χ^2^ (7, N = 1070) = 111.07, *p* < 0.001, Nagelkerke's R^2^ = 14.8.

**Table A2:** Results of the linear regression model with maximum heart rate as the outcome.

Model	B (95% CI)	*p*
Constant	156.070 (155.206, 156.933)	<0.001
β-blocker	−5.457 (−6.678, −4.236)	<0.001
Age	−1.017 (−1.088, −0.945)	<0.001
Female	0.448 (−1.533, 2.429)	0.657
Female × β-blocker	−1.690 (−4.492, 1.111)	0.237
Age × β-blocker	0.165 (0.064, 0.266)	0.001

B, unstandardized regression coefficients; CI, 95% confidence interval; linear regression model: F(5, 1064) = 286.01, *p* < 0.001, R^2^ = 0.57.

**Table A3:** Results of the linear regression model with maximum power as the outcome.

	B (95% CI)	*p*
Constant	206.659 (203.407, 209.912)	<0.001
β-blocker	−37.528 (−42.128, −32.929)	<0.001
Age	−2.717 (−2.988, −2.447)	<0.001
Female	−64.954 (−72.416, −57.493)	<0.001
Female × β-blocker	14.189 (3.637, 24.742)	0.008
Age × β-blocker	0.829 (0.447, 1.210)	<0.001

B, unstandardized regression coefficients; CI, 95% confidence interval; linear regression model: F(5, 1064) = 276.21, *p* < 0.001, R^2^ = 0.57.
